# TCP14 and TCP15 affect internode length and leaf shape in Arabidopsis

**DOI:** 10.1111/j.1365-313X.2011.04674.x

**Published:** 2011-07-21

**Authors:** Martin Kieffer, Vera Master, Richard Waites, Brendan Davies

**Affiliations:** 1 Centre for Plant Sciences, Faculty of Biological Sciences, University of LeedsLeeds LS2 9JT, UK; 2 Department of Biology, University of YorkPO Box 373, York YO10 5YW, UK

**Keywords:** TCP, transcription factor, development, morphometric analysis, *Arabidopsis thaliana*

## Abstract

TCP transcription factors constitute a small family of plant-specific bHLH-containing, DNA-binding proteins that have been implicated in the control of cell proliferation in plants. Despite the significant role that is likely to be played by genes that control cell division in the elaboration of plant architecture, functional analysis of this family by forward and reverse genetics has been hampered by genetic redundancy. Here we show that mutants in two related class I *TCP* genes display a range of growth-related phenotypes, consistent with their dynamic expression patterns; these phenotypes are enhanced in the double mutant. Together, the two genes influence plant stature by promoting cell division in young internodes. Reporter gene analysis and use of SRDX fusions suggested that *TCP14* and *TCP15* modulate cell proliferation in the developing leaf blade and specific floral tissues; a role that was not apparent in our phenotypic analysis of single or double mutants. However, when the relevant mutants were subjected to computer-aided morphological analysis of the leaves, the consequences of loss of either or both genes became obvious. The effects on cell proliferation of perturbing the function of *TCP14* and *TCP15* vary with tissue, as has been suggested for other TCP factors. These findings indicate that the precise elaboration of plant form is dependent on the cumulative influence of many TCP factors acting in a context-dependent fashion. The study highlights the need for advanced methods of phenotypic analysis in order to characterize phenotypes and to construct a dynamic model for TCP gene function.

## Introduction

The establishment of plant architecture and organ shape relies on complex cell proliferation and differentiation patterns, established in response to genetic and environmental cues (Ingram and Waites, 2006). The TCP family of plant-specific, non-canonical bHLH transcription factors (Cubas *et al.*, 1999; Martín-Trillo and Cubas, 2010) has been specifically linked to regulation of cell proliferation during development (Li *et al.*, 2005; Hervé*et al.*, 2009). Understanding the individual and combined roles and contributions of this transcription factor family is essential for establishment of a genetic model to describe the interaction between cell proliferation and spatial development.

Evolutionary studies have indicated that the TCP family has an ancient origin, as genes encoding TCP factors have been identified in pteridophytes, lycophytes, moss and some algal species, suggesting their likely appearance as between 800 and 650 million years ago (Navaud *et al.*, 2007). Indeed, representatives of the two distinct TCP sub-families found in Arabidopsis today (known as class I or TCPp, and class II or TCPc) are also found in the single-celled desmid algae *Cosmarium* spp. (Navaud *et al.*, 2007). These two TCP sub-families have been conserved and amplified through rounds of gene and genome duplication over millions of years to give rise to the extended *TCP* family of 24 genes in Arabidopsis (Martín-Trillo and Cubas, 2010).

Some form of functional analysis has been reported for ten of the eleven *TCP* genes of the class II (TCPc) sub-class identified in the Arabidopsis genome. Mutant phenotypes have been described for *TCP2*, *TCP4* and *TCP10* [epinastic cotyledons and slightly enlarged leaves (Schommer *et al.*, 2008)] and *TCP12* and *TCP18*, also known as *BRC2* and *BRC1* [control of shoot branching (Aguilar-Martínez *et al.*, 2007; Finlayson, 2007)]. Additional mutant phenotypes have been associated with *TCP4* [produces more leaves before flowering (Schommer *et al.*, 2008) and maternal effect embryo arrest (Pagnussat *et al.*, 2005)]. SRDX repression domain (Hiratsu *et al.*, 2003) fusions have been described for TCP2, TCP3, TCP4, TCP5, TCP10, TCP13, TCP17 and TCP24, and the results suggest redundant roles in regulation of lateral organ morphogenesis (Koyama *et al.*, 2007), and for *TCP1*, which appears to regulate the expression of a brassinosteroid synthesis gene (Guo *et al.*, 2010) and to play a role in leaf, petiole and stem elongation (Koyama *et al.*, 2010a). Lines that over-express the *jaw-D* microRNA, leading to a reduction in expression of five class II *TCP* genes, produce crinkly leaves (Palatnik *et al.*, 2003). The fact that several class II genes are regulated by the miRNA miR319 also led to the discovery that jasmonate biosynthesis and senescence are regulated by a subset of these TCP factors (Schommer *et al.*, 2008). When miR319 is over-expressed to down-regulate *TCP2*, *TCP3*, *TCP4*, *TCP10* and *TCP24* in a background in which *TCP5*, *TCP13* and *TCP17* are also down-regulated, large deeply lobed, serrated leaves are produced (Efroni *et al.*, 2008).

Class I (TCPp) sub-family members are more numerous in species analyzed to date, including Arabidopsis (Navaud *et al.*, 2007), but they are less well characterized. Mutant phenotypes have been reported for just two of the 13 Arabidopsis class I genes: *TCP21* (*CHE*), which shows enhanced activity of the circadian clock promoter *CCA1* (Pruneda-Paz *et al.*, 2009), and *TCP14*, which shows delayed germination (Tatematsu *et al.*, 2008a). In support of a more general link to circadian regulation, members of both class I (TCP11 and TCP15) and class II (TCP2 and TCP3) have been shown to participate in protein–protein interactions with various components of the circadian clock (Giraud *et al.*, 2010). Use of an RNAi approach has shown that *TCP16* plays a role in early-stage pollen development (Takeda *et al.*, 2006). Finally, VP16 activation domain and SRDX repression domain methods have indicated that TCP20 is involved in the regulation of cell division, growth and expansion (Hervé*et al.*, 2009).

Despite these apparently diverse phenotypes, class I and II TCP factors have been suggested to act antagonistically on plant cell division and growth, with class I factors promoting growth and class II factors inhibiting it (Li *et al.*, 2005). There is considerable evidence to support such a negative role for the class II genes. One of the founding members of the TCP family, *CYCLOIDEA* (*CYC*) in *Antirrhinum*, restricts the proliferation of cells within specific floral organs, as does its close relative *DICHOTOMA* (Luo *et al.*, 1996, 1999). Another founder member of the family, *TEOSINTE BRANCHED 1* (*tb1*) of *Zea mays*, restricts the proliferation of maize lateral branches (Doebley *et al.*, 1997), as do the *TCP18* and *TCP12* genes of Arabidopsis (Aguilar-Martínez *et al.*, 2007; Finlayson, 2007). Similarly, mutants of the *Antirrhinum* class II gene *CINCINNATA* show excessive proliferation at leaf margins, reflecting its normal role in dynamically restricting growth to produce a flat leaf surface (Nath *et al.*, 2003). These negative growth effects of class II TCP expression have been linked to a reduction in expression of cell-cycle markers such as histones and cyclins (Gaudin *et al.*, 2000; Nath *et al.*, 2003). In contrast, class I genes are thought to act positively on cell proliferation, although the evidence is more circumstantial. Two of the founding members of the TCP gene family, the rice class I genes *PCF1* and *PCF2*, were suggested to be activators of the proliferating cell nuclear antigen gene *PCNA* (Kosugi and Ohashi, 1997), and class I DNA-binding sites have been found to be over-represented in the promoters of growth and cell cycle-associated genes, including genes involved in ribosome biosynthesis and oxidative phosphorylation (Trémousaygue *et al.*, 2003; Li *et al.*, 2005; Tatematsu *et al.*, 2005, 2008b; Welchen and Gonzalez, 2006).

Here we describe the developmental roles of two closely related Arabidopsis class I TCP factors, TCP14 (At3g47620) and TCP15 (At1g69690). We show that the genes encoding these factors act redundantly to regulate plant stature by promoting cell proliferation in young internodes. Despite the fact that gene expression analysis revealed a complex and dynamic pattern of gene expression in developing leaves, a corresponding leaf development phenotype was not observed in the single or double mutants. However, when the mutants were subjected to computer-aided morphological analysis, the morphological effects of loss of either or both genes became obvious. SRDX fusion experiments confirmed the loss-of-function phenotypes, and further suggested that the effect of *TCP* gene expression on cell proliferation is dependent on the tissue context. This highlights the necessity for advanced methods of phenotypic analysis in unraveling the mechanisms underlying the elaboration of plant form. The fine-tuning of cell division required to generate specific forms is likely to be partially determined by the sum of TCP factor activity in each tissue.

## Results

### TCP14 and TCP15 redundantly regulate internode elongation

**Figure 1:**
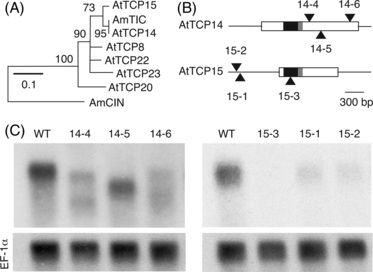
*TCP14* and *TCP15* insertion lines. (A) Unrooted phylogram of TCP14-related proteins. Neighbor-joining analysis of the TCP DNA-binding domain and adjacent CC domain that is important for protein dimerization (Kosugi and Ohashi, 1997; Cubas *et al.*, 1999) using PAUP4.0b (Swofford, 2002). Branches with bootstrap support >50% (1000 replicates) are indicated. The Arabidopsis TCP proteins included have been described by Cubas *et al.* (1999); the *Antirrhinum* proteins used were TIC (AJ580844) and CIN (AA043102) (used as outgroup). (B) *TCP14* and *TCP15* mono-exonic gene structure. The box represents the single exon. Black box, TCP domain; grey box, CC domain. T-DNA insertions are shown as black triangles. (C) Northern blot analysis of *TCP14* and *TCP15* transcripts in wild-type plants and mutants. Blotting with an elongation factor probe (EF-1α) was performed to show equal loading.

*TCP14* and *TCP15* are members of the class I sub-group of TCP factors that has 13 members in Arabidopsis (Martín-Trillo and Cubas, 2010) (Figure S1). These two *TCP* genes are the closest relatives of the *Antirrhinum* TCP factor TIC, which interacts with the organ boundary NAC-domain transcription factor CUPULIFORMIS (CUP) (Figure 1A) (Weir *et al.*, 2004). To determine the function of these two closely related genes in Arabidopsis, we analyzed a series of T-DNA insertion mutants. Three independent *TCP14* T-DNA insertion lines (*tcp14-4*, *tcp14-5* and *tcp14-6*, Figure 1B) were studied in detail, but no obvious phenotype was identified. Northern blot analysis showed that the T-DNA insertions in the three mutant lines caused a reduction in transcript level and resulted in production of truncated transcripts in all cases (Figure 1C). Hence, although these mutants do not represent null alleles, the *TCP14* transcripts are significantly altered. As all three insertions lie within the coding sequence, *TCP14* expression is likely to be compromised in each of these lines.

As the lack of visible phenotype in the three *TCP14* insertion lines could result from genetic redundancy with the closely related *TCP15* gene, we further analyzed three independent T-DNA insertion lines in which *TCP15* was disrupted (Figure 1A,B). Northern blot analysis showed that one T-DNA insertion (*tcp15-3*) destabilized the mutant transcript, and thus transcripts were not detected in this line (Figure 1C). The other two *TCP15* mutants (*tcp15-1* and *tcp15-2*), in which the insertions are located in the upstream region of the gene, induce a major reduction in transcript level. In contrast to the *tcp14* mutant lines, a phenotypic difference was observed when *tcp15* mutants were compared to wild-type (WT) control plants.

**Figure 2:**
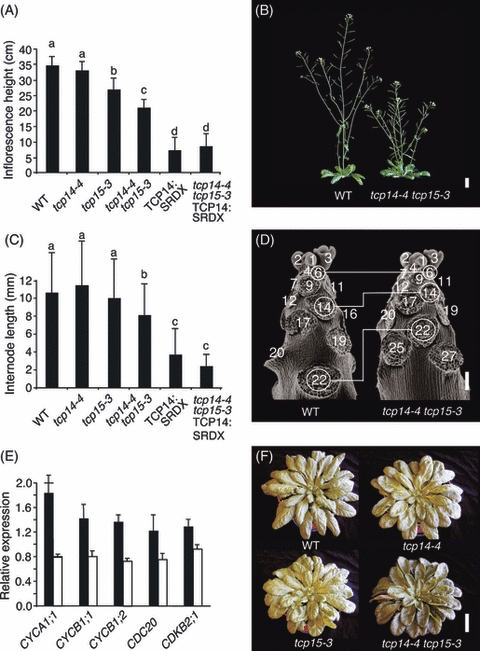
Mutant phenotypes. (A, B) Inflorescence heights of 42-day-old plants grown under long-day conditions: WT (Col-0), *tcp14-4*, *tcp15-3*, *tcp14-4 tcp15-3*, and *pTCP14:TCP14:SRDX* (annotated *TCP14:SRDX*) in WT and *tcp14-4 tcp15-3* backgrounds (63 < *n* < 170). Values are means and SD. (C, D) Inflorescence internode analysis. (c) Internode length of WT, single and double homozygous mutants, and *pTCP14: TCP14:SRDX* in WT and double mutant backgrounds (*n* = 100). Different letters above bars indicate statistically significant differences (Student's *t*-test, *P* < 0.001). Values are means and SD. (D) Scanning electron micrograph of dissected apices of WT (left) and *tcp14-4 tcp15-3* (right) inflorescences. Most flower primordia were removed (pedicel wounds visible). Primordia are numbered according to their initiation sequence. Some primordia of equivalent age are indicated by circled numbers connected by lines to highlight differences in internode elongation. (E) Relative expression levels of *CYCA1;1*, *CYCB1;1*, *CYCB1;2*, *CDC20* and *CDKB2;1* in dissected apices of WT (black bar) and *tcp14-4 tcp15-3* mutant (white bar) monitored by real-time quantitative PCR. Each bar represents the mean of nine replicates from three independent plant samples relative to EF-1α. Values are means and SD. (F) Rosettes of WT (Col-0), *tcp14-4*, *tcp15-3* and *tcp14-4 tcp15-3* grown under short-day conditions. Sacle bars = 1 cm (B), 50 μm (D) and 2 cm (F).

The *tcp15-3* mutant showed a mild but highly significant reduction in inflorescence height (Student's *t-*test, *P* < 0.001) (Figure 2A). Fruit pedicel length was also reduced in this mutant. To test whether *TCP15* and *TCP14* exhibit redundancy, a double mutant was constructed. The *tcp14-4 tcp15-3* double mutant showed a further significant reduction in inflorescence height (Figure 2A,B) and pedicel length (Figure S2C) (Student's *t-*test, *P* < 0.001). These defects were seen in all four allelic combinations tested, including all three *TCP15* alleles and two of the *TCP14* alleles (Figure S2A). Furthermore, the reduction in inflorescence height and pedicel length was complemented by expression of *TCP14* under the control of its native promoter (Figure S2B–D). The number of leaves produced at the floral transition did not differ significantly between WT (14.1 ± 2.0, *n* = 169) and the *tcp14 tcp15* double mutant plants (13.2 ± 1.6, *n* = 131), indicating that the phenotype is unlikely to be caused by nutritional limitation. Biometric analysis of inflorescence stems revealed that internode elongation was significantly reduced in the double mutants (Student's *t-*test, *P* < 0.001) (Figure 2C). Although individual internode sizes vary widely within the stems of both wild-type and mutant lines, the cumulative effect of the shorter internodes in the double mutant is a significant reduction of the overall inflorescence stem length (Figure 2A–C).

To determine how early the reduced internode defect occurs, scanning electron micrographs of shoot apices were analyzed. These showed that internode elongation is reduced in the double mutant from early stages (Figure 2D). To assess cell proliferation in the inflorescence, we analyzed the expression of five effectors of cell division (G_2_/M and mitosis markers) by quantitative real-time PCR. These genes were selected for their high periodicity of expression during the cell cycle (Menges *et al.*, 2005). The expression of all five genes, including *CYCLIN B1;1*, a G_2_/M marker previously reported to be a target of the class I factor TCP20 (Li *et al.*, 2005), was reduced in mutant apices relative to the wild-type (Figure 2E). These results show that *TCP14* and *TCP15* act redundantly to promote cell proliferation in young stem internodes, and together modulate plant stature. Despite careful analysis, we were unable to discern any other phenotypic consequences in the double mutant, other than an effect on leaf curling in mature leaves of plants grown under short-day conditions (Figure 1F). Equivalent leaves from WT, *tcp14-4* and *tcp15-3* plants all showed downward (revolute) curling at the leaf margins, leaves on the *tcp14-4 tcp15-3* double mutant were upwardly curled (involute) at their margins. These differences were only observed in mature leaves; no obvious phenotypic consequences were observed in young and developing leaves of the single or double mutants. Notwithstanding this, the switch from revolute to involute curling that is apparent in mature leaves strongly suggests that, in addition to their role in promoting cell proliferation in young internodes, these genes are also involved in regulating cell proliferation within the leaf, although it is not apparent whether this effect is positive or negative.

### *TCP14* and *TCP15* are dynamically expressed in young proliferating tissues

**Figure 3:**
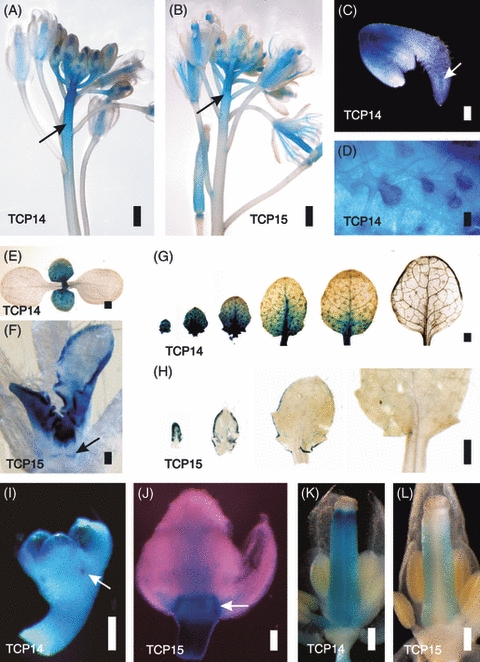
Expression pattern of *TCP14* (*pTCP14:TCP14:GUS*) and *TCP15* (*pTCP15:TCP15:GUS*). (A, B) Mature inflorescences. (C) Young germinating embryo of *pTCP14:TCP14:GUS.* The arrow shows a stained radicle stele). (D) *TCP14* expression in leaf trichomes. (E–H) Expression pattern of *TCP14* and *TCP15* in young seedlings at 7 days after germination (E, F) and in leaves (at 20 days after germination) displayed according to their sequence of initiation (left to right) (G, H). (I, J) *TCP14* expression in a stage 5 flower (arrowhead indicates staining at sepal/sepal boundary), and *TCP15* expression in a stage 8 flower (arrowhead indicates staining at sepal/receptacle boundary). (K, L) Expression of each gene in the gynecium of partly dissected flowers (stage 11). Scale bars = 1.5 mm (A, B, E), 100 μm (C), 50 μm (D); 30 μm (F, I, J), 1 mm (G, H) and 250 μm (K, L).

Attempts to visualize the spatial and temporal expression of *TCP14* and *TCP15* by *in situ* hybridization were unsuccessful, probably due to the low level of expression of these genes. In order to study *TCP14* and *TCP15* expression, we therefore constructed lines expressing translational fusions with the GUS reporter gene, under the control of their native promoters (1.75 and 1.92 kb, respectively). Thirty lines were studied for each gene, and showed distinct but related GUS staining patterns. As expected from the mutant analysis, both genes are expressed in all internodes of the young inflorescence stems and in young flower pedicels, and their expression gradually ceases as the tissues mature (Figure 3A,B). In young seedlings, *TCP14* is expressed at the base of the cotyledons, in the stele of the radicle and the hypocotyl (Figure 3C). *TCP14* is also expressed at low levels in the vegetative shoot apical meristem, and both genes are expressed in leaf primordia (Figure 3E,F). *TCP15* is mostly expressed in primordial leaf margins (Figure 3F,H), while *TCP14* is expressed more widely in the leaf blade (Figure 3E,G), including young trichomes (Figure 3D) and developing leaf vascular bundles (Figure 3G). As leaves mature, *TCP14/15* gene expression in leaves is repressed in a apical to basal (basiplastic) manner (Figure 3G,H). No expression was detected in inflorescence apical meristems or the youngest floral primordia. However, by stage 3, expression of both genes is detected in the whole flower, particularly in sepals (Figure 3I,J). In older flowers, expression is observed in young stamens and carpel valves (Figure 3K,L and Figure S3). As the gynecium matures, expression in the valves is gradually reduced. We also detected strong foci of *TCP14* expression at the sepal/sepal boundaries and of *TCP15* expression at the pedicel/sepal boundary (Figure 3I,J). *TCP15* expression was also detected at the stem/leaf boundary (Figure 3F). The observed GUS patterns are broadly consistent with publicly available microarray data (Figure S4) (Zimmermann *et al.*, 2004).

Expression of both genes is dynamic in young stems, leaves and carpel valves, and corresponds with developmental stages defined by widespread but highly regulated cell proliferation. Expression in stems is consistent with the role of TCP14 and TCP15 in promoting cell proliferation in young internodes. In leaves, the genes are also expressed in vascular bundles, in which cell proliferation remains temporarily active after the main phase of blade proliferation (Donnelly *et al.*, 1999). However, *TCP14* and *TCP15* are also strongly expressed at the boundaries of leaves and sepals, where cell division is highly restricted (Breuil-Broyer *et al.*, 2004; Laufs *et al.*, 2004), suggesting that TCP14 and TCP15 may not be capable of stimulating cell proliferation in every tissue. Despite the clear, dynamic expression patterns of both genes throughout the course of leaf development, we did not observe any phenotypic consequence arising from single or double *tcp14 tcp15* mutants with respect to leaf shape or size, other than the late effect on leaf curling in mature leaves (Figure 2F). Furthermore, leaf cells in *tcp14*, *tcp15* and *tcp14 tcp15* mutants showed the same size and morphology as WT controls.

### Quantitative imaging shows that TCP14 and TCP15 modulate leaf blade expansion

**Figure 4:**
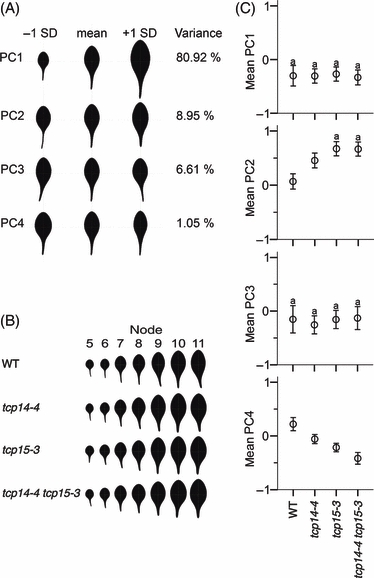
Analysis of leaf size and shape in WT, *tcp14-4*, *tcp15-3* and *tcp14-4 tcp15-3* plants. (A) Leaf size and shape according to variation along the first four PCs for the Arabidopsis leaf-point model of 1500 leaves. The mean leaf is shown, together with leaves for 1 SD either side of the mean leaf for each PC. The variance for each PC is indicated as a percentage. Together, the four PCs represent 97.53% of the total variation in leaf shape and size within the sample. (B) Leaf point models for leaves in node positions 5–11 calculated from mean PC1, PC2, PC3 and PC4 values for each leaf for WT, *tcp14-4*, *tcp15-3* and *tcp14-4 tcp15-3* plants, representing the mean size and shape of the leaves for each line. (C) Mean PC scores for all leaves from nodes 5–11 for WT, *tcp14-4*, *tcp15-3* and *tcp14-4 tcp15-3*. Error bars represent 95% confidence intervals. The letter ‘a’ indicates that there is no significant difference between genotypes.

The complex, dynamic expression patterns of *TCP14* and *TCP15* during leaf development, combined with their role in promoting cell division in internodes, suggested that our failure to observe an early leaf development phenotype could be due to the inherent difficulty in discriminating between leaf morphologies in developing leaves. We used a quantitative imaging approach, LeafAnalyser (Weight *et al.*, 2008), to address this limitation. We first defined the natural variation in Arabidopsis leaf shape by constructing a library of 1500 leaves from defined nodes of ten Arabidopsis accessions. Using LeafAnalyser, all leaves were aligned by rotation until the leaf tip was vertically above the centre-most point of the leaf, and by translation until the centre-most point of each leaf was in the same position. LeafAnalyser was then used to position 48 landmarks around the margin of each leaf, and derive a leaf-point model from the 96 co-ordinate values these landmarks generated. A principal component analysis was then applied to the dataset of 1500 leaf-point models using LeafAnalyser. The first four principal components (PCs) accounted for 97.53% of the variance in leaf shape and size, indicating that there was significant correlation of the variation in position between the leaf margin landmarks. The variation in leaf shape and size captured along PC1, PC2, PC3 and PC4 is shown as standard deviations from the mean leaf in Figure 4(A). Variation along PC1 accounts for 80.92% of the natural variance in leaf shape and size, and a higher value of PC1 corresponds to an increase in size. Variation in leaf shape is captured by PC2, and accounts for 8.95% of the total variation. Curvature of the leaf, with the petiole aligned either to the right or the left, is captured by PC3 and accounts for 6.61% of the total variance. PC4 captures the aspect ratio (leaf length:leaf width), and accounts for 1.05% of the variance.

In order to test whether there were differences in leaf size and shape between WT, *tcp14-4*, *tcp15-3*, and *tcp14-4 tcp15-3* plants, we used LeafAnalyser to generate leaf-point models for leaves of nodes 5–11 from 20 plants of each line. We then applied the principal component analysis from the Arabidopsis accessions to these leaf-point models, and generated standardized PC scores for each leaf (see Experimental Procedures for a more detailed explanation). Using these values, we reconstructed mean leaves (Figure 4B) and calculated mean PC values (Figure 4C) for each line, which suggested that leaf shape did indeed vary. We used a one-way anova to test whether the mean PC scores differed significantly, explaining the differences between lines. Along PC2, *tcp14-4*, *tcp15-3* and *tcp14-4 tcp15-3* differed significantly from WT (*P* < 0.001), and *tcp14-4* differed significantly from *tcp15-3* and *tcp14-4 tcp15-3* (*P* < 0.05) (Figure 4C). Along PC4, *tcp14-4*, *tcp15-3* and *tcp14-4 tcp15-3* differed significantly from WT (*P* < 0.001), *tcp14-4* differed significantly from *tcp15-3* (*P* < 0.05) and *tcp14-4 tcp15-3* (*P* < 0.001), and *tcp15-3* differed significantly from *tcp14-4 tcp15-3* (*P* < 0.05) (Figure 4C). No significant differences were found for mean PC1 and PC3 values between these lines.

These differences, although impossible to visualize by eye, are readily detectable using a quantitative imaging approach. The leaves show blade shape defects in the order WT >*tcp14*>*tcp15*>*tcp14 tcp15*, and these mirror the severity of the observed plant stature phenotype. The mutants show a shift to a higher PC2 value and a lower PC4 value than wild-type. The direction and magnitude of the associated cumulative changes in leaf margin landmark positions are shown in Figure S5. The observed changes in PC2 and PC4 together capture a distal shift of the centre of the leaf, producing a leaf that is broader towards the base. The petiole shape is also altered, becoming broader and shorter. Taken together, these results show that, consistent with their dynamic expression patterns during leaf development, TCP14 and TCP15 modulate overall leaf shape. Although one might predict that disrupting genes that affect cell proliferation would lead to altered leaf size, the observed consequence is more subtle than this. Loss of function of either or both genes specifically affects leaf shape and aspect ratio. Even though the expression of *TCP15* in leaves is predominantly confined to the margins, *tcp15* mutants have a more severe leaf shape defect than *tcp14* mutants. The larger influence of *TCP15* on leaf shape could be due to factors such as the role of the margin in defining shape, signaling from the margin to the blade, some *TCP15* expression in the blade, or further redundancy that affects *TCP14* more than *TCP15*. Overall, these findings suggest that organ shape is defined by rigorous control of cell proliferation in specific cells and tissues, mediated by expression of a combination of TCP factors. Individual *tcp* mutants are likely to have subtle phenotypes that require the use of computerized phenotyping approaches for identification. However, shape is determined in part by the combination of TCP action throughout development of an organ.

### *TCP14/15* genes repress cell proliferation in leaf and floral tissues

**Figure 5:**
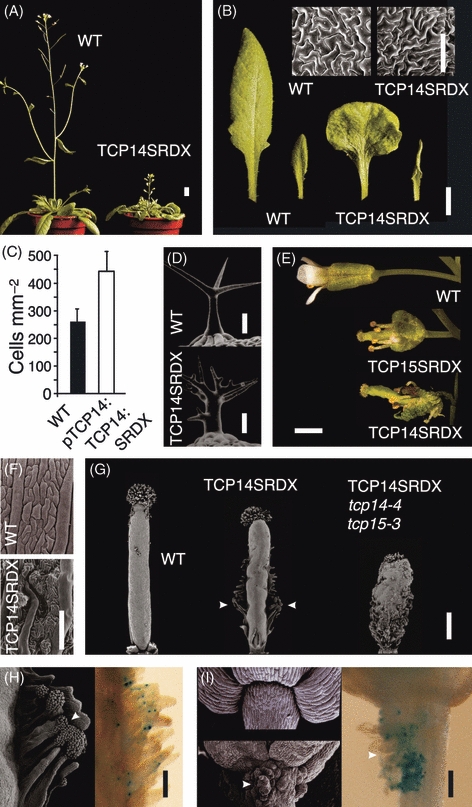
Phenotype of *TCP14* EAR repression domain fusion lines (*pTCP14:TCP14:SRDX*). (A) WT and *pTCP14:TCP14:SRDX* plants. (B) Leaves from WT and *pTCP14:TCP14:SRDX* plants at young (right) and mature (left) stages of development. The inset panels show scanning electron micrographs of the adaxial epidermis of mature leaves of WT and *pTCP14:TCP14:SRDX*. (C) Density of adaxial epidermal cells of mature leaves from WT (black bar) and *pTCP14:TCP14:SRDX* (white bar). Values are means and SD. (D) Trichomes from WT and *pTCP14:TCP14:SRDX* leaves. (E) Mature wild-type flower (top), *pTCP15:TCP15:SRDX* flower (middle) and *pTCP14:TCP14:SRDX* flower (bottom). (F) Scanning electron micrographs of sepal adaxial epidermis of wild-type and *pTCP14:TCP14:SRDX* flowers. (G) Gynecium micrographs (carpel valve view) of dissected mature flowers from WT, and *pTCP14:TCP14:SRDX* in WT and *tcp14-4 tcp15-3* backgrounds (arrowheads show tissue outgrowth). (H) Close-up of the carpel/replum boundary tissue outgrowth: micrograph (left) (arrowheads indicate stigmatic papillae); GUS staining in a *Cyc1At::GUS pTCP14:TCP14:SRDX* line (right). (I) Scanning electron micrographs of WT (top left) and *pTCP14:TCP14:SRDX* (bottom left) flower receptacles. Right: GUS staining of a *Cyc1At::GUS pTCP 14:TCP14:SRDX* flower receptacle (arrowheads indicate tissue outgrowth). Scale bars = 1 cm (A, B), 100 μm (inset to B, F, H, I), 50 μm (D), 1 mm (E) and 0.5 mm (G).

If shape is influenced by multiple TCP factors, the mutant phenotypes described here should be enhanced in backgrounds in which expression of related class I *TCP* genes is also compromised. The *TCP14* and *TCP15* genes belong to a small sub-clade that includes three other genes (*TCP8*, *TCP22* and *TCP23*, Figure 1A) that are clustered with *TCP15* on chromosome 1. This is often the case for members of multi-gene families, and makes combining multiple mutants a difficult task. To overcome this, we constructed lines expressing *TCP14* fused to the short EAR motif (*TCP14:SRDX*). This approach, which has been extensively used to study the function of class II TCP factors (Koyama *et al.*, 2007, 2010a; Guo *et al.*, 2010), relies on the ability of the EAR motif to convert transcriptional factors into dominant repressors to produce loss-of-function phenotypes even in the presence of redundant genes (Hiratsu *et al.*, 2003). Expression of *TCP14:SRDX* under the control of its native promoter (*pTCP14:TCP14:SRDX*) produced plants with a severe reduction in stem and pedicel elongation (81 of 82 primary transformants) (Figure 5A). This reduction was even stronger than that seen in the *tcp14-4 tcp15-3* double mutant (Figure 2A,C), validating the suitability of the approach and confirming the influence of redundancy on the double mutant.

In addition to even shorter inflorescence stems and pedicels than in the *tcp14 tcp15* double mutants, *pTCP14:TCP14:SRDX* lines showed conspicuous leaf defects. The reduction in tip elongation and broadening of the leaf base, which could only previously be distinguished in the mutants by using LeafAnalyser, could now be observed (Figure 5B). Furthermore, the margins of young *pTCP14:TCP14:SRDX* leaves are initially small, narrow and upwardly curled (involute), in contrast to WT leaves, which are first flat and then become gradually curled downward (revolute) at later stages (Figure 5B). This effect mirrors the phenotype observed in mature leaves of the *tcp14 tcp15* double mutant grown under short-day conditions (Figure 2F), but occurs from early stages of leaf development in the *pTCP14:TCP14:SRDX* lines. Therefore, all three aspects of the *tcp14 tcp15* double mutant phenotype, i.e. reduced internode and pedicel length, altered leaf shape and involute leaf curling, are enhanced in the *pTCP14:TCP14:SRDX* plants. Similar phenotypes were previously reported in mutant and transgenic lines with altered proliferation of leaf blade cells (Kim *et al.*, 1998; Serrano-Cartagena *et al.*, 2000; Bemis and Torii, 2007). To test this hypothesis, we analyzed leaf adaxial epidermal cell density using scanning electron micrographs of mature WT and *pTCP14:TCP14:SRDX* leaves (Figure 5B). The transgenic lines showed a twofold increase in cell density compared to WT leaves (Student's *t-*test, *P* = 0.0012) (Figure 5C). Therefore, *pTCP14:TCP14:SRDX* leaves are characterized by an excess of cell proliferation in a given area, and show enhancements of the shape and curling phenotypes observed in the *tcp14 tcp15* double mutant. Most mature *pTCP14:TCP14:SRDX* leaf margins also bear excessively branched trichomes (Figure 5D), a defect that is often associated with abnormally high ploidy (excessive number of endo-reduplication cycles) (Schellmann and Hülskamp, 2005).

In association with a severe reduction in flower pedicel length, *pTCP14:TCP14:SRDX* plants also showed a general alteration of floral organ development, including strong sepal, petal and carpel defects (Figure 5E). Similar phenotypic effects were also observed in transgenic plants expressing a TCP15–SRDX fusion (*pTCP15:TCP15:SRDX*). The sepal blades of the transgenic plants showed a higher cell density than in WT tissues (Figure 5F), suggesting a similar increase in cell proliferation to that observed in leaves. Carpels of *pTCP14:TCP14:SRDX* plants showed striking tissue outgrowths along the lower boundaries of the valves and the gynophore and the lateral lower/medial boundaries with the replum (Figure 5G,H). The outgrowths often terminate with stigmatic papillae (Figure 5H) and elongate perpendicular to the direction of gynecium elongation. Ectopic tissue proliferation was also observed on the flower receptacle in 5–10% of the transformants (Figure 5I). Both ectopic tissue proliferation on the flower receptacle and carpel valve boundaries were associated with mitotic cells expressing *CYCLINB1;2* (Figure 5H,I). Although the *pTCP14:TCP14:SRDX* plants displayed strongly enhanced versions of the *tcp14 tcp15* double mutant phenotype, even stronger phenotypes were observed when *pTCP14:TCP14:SRDX* was introduced into the *tcp14-4 tcp15-3* double mutant background (Figures 2C and 5G), suggesting that some residual TCP14 activity is still present in the TCP14:SRDX lines. The fact that *pTCP14:TCP14:SRDX* lines display enhanced versions of all observed aspects of the *tcp14 tcp15* double mutant phenotype whilst showing reduced cell proliferation in internodes and enhanced cell proliferation in leaves and floral tissues suggests that the influence of TCP factors on cell proliferation is context-dependent.

## Discussion

### The developmental roles of TCP14 and TCP15

We show here that TCP14 and TCP15 redundantly affect plant stature by promoting cell proliferation in young internodes. This role is consistent with their expression patterns in the stem, which peaks in young internodes and decreases later. The effect on stature is most easily observed in the *tcp14 tdp15* double mutant or *TCP14:SRDX* lines, highlighting the influence of genetic redundancy. Plant stature is an important agronomic trait. The rice orthologs of *TCP14* and *TCP15* are *OsTCP5* (Os02g51280) and *OsTCP12* (Os06g12230) (Figure S1). It has previously been reported that expression of *OsTCP5* is undetectable by RT-PCR, whereas *OsTCP12* is predominantly expressed in the stem (Yao *et al.*, 2007). It is therefore possible that *OsTCP12* could be a promising target for reducing the stature of rice plants.

TCP14 and TCP15 also play a role in elaboration of leaf shape. Both genes are first expressed in the whole leaf, but expression is gradually arrested in an apical to basal manner that mimics the main basiplastic wave of cell proliferation arrest. Loss-of-function mutants and *TCP14:SRDX* lines show an alteration in leaf shape, with the leaf becoming less elongated at the tip and broader at the base, and also showing a tendency to reversal of the natural revolute curling to involute curling. The shape differences observed in these lines are associated with an increase in cell density, suggesting that TCP14 and TCP15 act as repressors of cell proliferation in the developing leaf. The class II *TCP* gene *CIN* was also reported to act as a repressor of cell proliferation in leaves (Nath *et al.*, 2003). More recently, another class II *TCP* gene, *TCP1*, was also investigated, and, although no phenotype was observed using an RNAi approach, dwarfing and shorter leaves and petioles were seen using an SRDX fusion strategy (Koyama *et al.*, 2010a). The spatial expression of *TCP1* (Koyama *et al.*, 2010a) is remarkable when compared to the expression of *TCP14* and *TCP15* presented here, as it appears to be complementary. Hence *TCP1* and *TCP14*/*TCP15*, which promote longitudinal proliferation of the stem, are expressed in mutually exclusive zones, with *TCP14/TCP15* being confined to young internodes and *TCP1* to older internodes. Similarly, in leaves, *TCP1* expression primarily occurs at the tip, and *TCP14/TCP15* expression is confined to the base.

### The influence of TCP14 and TCP15 on cell proliferation is context-dependent

The classical view of the role of TCP transcription factors on cell proliferation has been that class I factors promote proliferation and class II factors repress it. Our results, and those of others, challenge this view. Disruption of *TCP14* and *TCP15* affects plant stature by reducing stem internode elongation. This defect is associated with a reduction of cell proliferation, characterized by reduced expression levels of a range of effectors of cell division, including cyclins and cyclin-dependent kinases. This suggests that TCP14 and TCP15 promote cell proliferation in young internodes. In agreement with these findings, TCP14 was recently reported to also promote cell proliferation in the embryo during germination (Tatematsu *et al.*, 2008a). Expression of TCP14 fused to the EAR domain produces a stronger version of the internode elongation phenotype. *tcp14 tcp15* mutants additionally show mild leaf defects that are exaggerated in *pTCP14:TCP14:SRDX* lines, which also show new floral phenotypes. In the *pTCP14:TCP14:SRDX* lines, the leaves are broader at the base, and analysis of leaf cell density shows that cell proliferation is increased, in contrast to the reduction observed in the double mutant stem. During the cell cycle, cyclin B1s (*CYCB1*) are the main activators promoting G_2_/M progression (reviewed by Ramirez-Parra *et al.*, 2005). *CYCB1;2* expression is reduced in young stems of *tcp14 tcp15* double mutants. However, the floral organs of *TCP14:SRDX* plants show increases in cell proliferation in specific sepal, carpel and receptacle tissues. These results indicate that *TCP14* and *TCP15* can promote or repress cell proliferation in a tissue-dependent manner. Over-expression of VP16 and SRDX fusions of another class I *TCP* gene, *TCP20*, has previously been cited as evidence for context-dependent function (Hervé*et al.*, 2009), as has expression of a microRNA-resistant form of the class II *TCP* gene, *TCP2* (Palatnik *et al.*, 2003). It is therefore possible that the effect of many or all TCP factors on cell proliferation varies with tissue and developmental stage. Taken together, these findings suggest a less rigid and more dynamic division of the roles of activation and repression of cell proliferation by both class I and class II TCP factors. The specific consequences of *TCP* gene expression on cell proliferation are likely to be determined by the developmental environment in which they are expressed. Although TCP14 and TCP15 can either promote or repress cell proliferation, depending on the developmental context, the fact that the *TCP14:SRDX* plants show enhancement of all aspects of the loss-of-function mutant phenotype suggests that TCP14 normally functions as an activator of target gene expression.

Cyclins activate cyclin-dependent kinases (CDKs), which regulate progression through mitosis. The transcript level of *CDKB1;2* is reduced in young *tcp14-4 tcp15-3* inflorescence stems. The expression of *CDKB1;1*, a gene closely related to *CDKB1;2*, peaks in G_2_ and regulates the balance between cell proliferation and endo-reduplication (Boudolf *et al.*, 2004). In *pTCP14:TCP14:SRDX* lines, leaves have highly branched trichomes, a phenotype that has been reported as a marker of abnormally high ploidy (Schellmann and Hülskamp, 2005). This phenotype may reflect premature entry into the endo-reduplication phase of trichome development. However, the increase in cell proliferation observed in the leaf epidermis indicates that entry into the endo-reduplication phase is delayed. These phenotypes suggest an unusual combination of increased cell proliferation in the leaf epidermis with possible promotion of endocycles in trichomes, as though disruption of the *TCP14/15* genes can promote mitotic or endo-reduplication cycles depending on the cellular context. Such defects were previously reported in hypocotyls of lines over-expressing the E2Fa–DPa complex. In these plants, hypocotyl cells underwent endo-reduplication if cell division had finished, and additional rounds of cell division if not (De Veylder *et al.*, 2002). The range of alterations observed in mutant and SRDX fusion lines suggests that the *TCP* genes modulate cell proliferation and differentiation in a cell- and tissue-dependent manner, possibly by affecting the duration of cell proliferation or the endo-reduplication phase.

### Genetic redundancy in the *TCP* gene family

The *TCP* gene family has been described as showing extreme genetic redundancy (Koyama *et al.*, 2010b) due to the difficulty in obtaining mutant phenotypes. Here we show that visualization of phenotypes can be facilitated by quantitative imaging approaches. We suggest that a sufficiently discriminatory quantitative imaging approach will show that more *TCP* single mutants have observable phenotypes. Genetic redundancy has been circumvented in the study of many Arabidopsis TCP genes by use of the SRDX repression system (Hiratsu *et al.*, 2003; Koyama *et al.*, 2007, 2010a; Guo *et al.*, 2010), although only one class I *TCP* gene has been studied in this way (Hervé*et al.*, 2009). The specific enhancement of all aspects of the single and double mutant phenotypes observed in our *TCP14:SRDX* lines strongly supports the validity of this approach. The increased severity of the phenotypes of the *TCP14:SRDX* lines compared to the *tcp14 tcp15* double mutant is also consistent with genetic redundancy extending beyond these two genes.

Based on the phenotypes observed for the single and double *tcp14* and *tcp15* mutants, we suggest that the reason for the lack of observed phenotypes in this family is that the TCP factors individually exert a subtle, dynamic and context-dependent effect on cell proliferation. The overall form of the plant is therefore generated by the sum of a large number of individually small influences on cell division, each of which is independently regulated. Furthermore, there is probably a strong requirement for coordination of regulatory signals. The involute leaf curling, seen at late stages in the *tcp14 tcp15* double mutant and from early stages in *TCP14:SRDX* lines, is probably caused by failure to compensate for localized increases in cell proliferation, resulting in inability to produce a flat leaf blade. This also means that predicting the effect of individual mutations on plant architecture will be impossible without a dynamic model that captures the full array of inputs. The difficulty of scaling up changes in leaf growth parameters to the whole-leaf or whole-plant scale has been discussed previously (Granier and Tardieu, 2009). In this context, quantitative imaging approaches are essential for understanding the relative and combined contributions of the individual genes. Such phenotyping methods could also address more widespread concerns regarding the prevalence of genetic redundancy when analysing gene function by reverse genetics (Bouché and Bouchez, 2001).

## Experimental Procedures

### Plant material and growth conditions

The *tcp15-3* mutant is line Salk_011491 (Alonso and Stepanova, 2003), and the *tcp15-1* and *tcp14-6* mutants are lines SAIL_682_F07 and SAIL_1145_H03 (Sessions *et al.*, 2002). *tcp14-4*, *tcp14-5* and *tcp15-2* were isolated by screening the T-DNA alpha population of the Arabidopsis knockout facility at the University of Wisconsin-Madison (Krysan *et al.*, 1999). *tcp14-5* also shows T-DNA sequence-induced silencing of *APETALA3* (Dinneny *et al.*, 2004). Before double mutant construction, *tcp14-4* was backcrossed four times in the Columbia background. All mutants were genotyped by PCR: primer 13R1 (5′-GTTGTTGATGATGATGTCTCTGTG-3′) was used in combination with primers JL-202 (5′-CATTTTATAATAACGCTGCGGACATCTAC-3′) for both *tcp14-4* and *tcp14-5* mutants and primer LB1 (5′-GCCTTTTCAGAAATGGATAAATAGCCTTGCTTCC-3′) for the *tcp14-6* mutant to generate DNA fragments of approximately 820, 630 and 300 bp, respectively. A WT band (1210 bp) was obtained using primers 13R1 and 13F1 (5′-GACGACAACCATCAACAACAACCTTC-3′). Use of primer 14R1 (5′-GGTTTTGCTGGTTGTTGTTATGGTTC-3′) and primer Lba1 (5'-ATGGTTCACGTAGTGGGCCATC-3') generated a *tcp15-3* mutant band (990 bp), and use of primer 14R1 with primer 14F1 (5′-GCCATCATCTCTACTACTTCCGAACCTAAC-3′) generated a 832 bp WT band. Use of primer 14F2 (5′-TGTATGTCAATGAGATAACTCCAATGGTG-3′) with LB1, JL-202 or 14R2 (5′-TGGTTCTGCTTGTTGGAGTAGCCAC-3′), respectively, produced *tcp15-1*, *tcp15-2* and WT bands of 550, 540 and 1266 bp. Plant transformation was performed using the method described by Clough and Bent (1998), and 100 primary transformants were identified and analyzed for most experiments. The GUS assay was performed as described by Jefferson *et al.* (1987). Plants were grown in Sanyo growth chambers (Sanyo, http://www.sanyo-biomedical.co.uk) at 20°C under long-day conditions (16 h, 200 μmol m^−2^ sec^−1^, 60% humidity).

### Vector construction

Mutant complementation was accomplished by transforming *tcp14-4 tcp15-3* plants with pGreenII0229 (Hellens *et al.*, 2000) carrying a 4.5 kb PCR fragment covering the *TCP14* promoter (1.9 kb), coding sequence and 3′ sequence (1.2 kb). The *pTCP14:TCP14:GUS* and *pTCP15:TCP15:GUS* reporters were constructed by cloning fragments encompassing the intronless *TCP14* and *TCP15* genes and their promoters (1.75 and 1.92 kb, respectively) using Gateway® technology (Invitrogen, http://www.invitrogen.com/) into the pJawohl11-GW-GUS vector (a derivative of pBIN19 kindly provided by Bekir Ülker, Department of Plant–Microbe Interactions Max Planck Institute for Plant Breeding Research, Cologne, Germany). For the *pTCP14:TCP14:SRDX* plasmid, pFP101 was digested with *Hin*dIII*/Xba*I to remove the 2x35S PRO cassette. Gateway cassette B was then ligated into the Klenow-filled *Hin*dIII*/Xba*I sites. The *TCP14* promoter and coding sequence was amplified by PCR, and a C-terminal EAR domain (SRDX) (Hiratsu *et al.*, 2003) was added by nested PCR before Gateway cloning into the modified pFP101 vector.

### RNA analysis

RNA was extracted using an RNeasy plant mini kit (Qiagen, http://www.qiagen.com/) and treated with DNase I (http://www.gehealthcare.com). RNA concentration and quality were assessed using an Agilent 2100 Bioanalyzer with an RNA Nano chip (Agilent Technologies, http://www.agilent.com). First-strand cDNA synthesis was performed using Omniscript® reverse transcriptase (Qiagen) with 1 μg of total RNA for each reaction. First-strand cDNAs were purified through a QIAquick® PCR purification column (Qiagen) before real-time quantitative PCR. Real-time quantitative PCR was preformed using IQ™ SYBR® Green Supermix (Bio-Rad, http://www.bio-rad.com/) in a 25 μl reaction using an IQ™ 4 real-time PCR machine (Bio-Rad). The following primer pairs were used: CYCA1;1F (5′-AAAGCGATGGAGTTGAGAGG-3′) and CYCA1;1R (5′-GTGGGATTACGGATGGACAA-3′); CYCB1;1F (5′-CTGATCCTGGTGGAGTGGTT-3′) and CYCB1;1R (5′-CATACTTGGCCGACATGAGA-3′); CYCB1;2F (5′-CTTGCTGAATTGGGGATGAT-3′) and CYCB1;2R (5′-CAGGGGACTTGTTCAATGAG-3′); CDC20F (5′-ATGGATGCAGGTTTGAATCG-3′) and CDC20R (5′-AGTGAGGGCAAAGTGAGCAT-3′); CDKB2;1F (5′-ATCTCCATTTTGCGAATGCT-3′) and CDKB2;1R (5′-GGAATGTTCTTGCCAGTGCTA-3′). Expression levels were normalized against elongation factor 1α, and the mean of nine replicates from three independent plant samples was calculated.

### Leaf shape analysis and models

Leaves were collected from WT (*n* = 20), *tcp14-4* (*n* = 20), *tcp15-3* (*n* = 20) and *tcp14-4 tcp15-3* (*n* = 20) plants after the first flowers had opened. Each leaf was removed, flattened between acetate overhead projector sheets, and scanned using a Hewlett-Packard Scanjet 4370 scanner (http://www.hp.com) at a resolution of 300 dpi. The leaves were processed using LeafAnaylser software (Weight *et al.*, 2008; http://leafanalyser.openillusionist.org.uk/). PC values for each leaf were obtained by multiplying the leaf-point models by the eigenvector matrix generated by LeafAnalyser from 1500 leaves from ten Arabidopsis accessions showing variation in leaf size and shape, which had been processed by LeafAnalyser in the same way. The values were scaled by dividing each value by the corresponding standard deviation for that PC, which had been defined as the square root of the eigenvalues generated by LeafAnalyser. Leaf point models representing mean leaves for node positions 5–11 were derived from the mean leaf by applying the relevant eigenvectors and the mean of the standard deviation for each PC determined for each leaf.

### Microscopy

Scanning electron microscopy samples were analyzed as previously described (Kieffer *et al.*, 2006).

## SUPPORTING INFORMATION

**Figure S1.** Phylogenetic analysis of the Arabidopsis and rice TCP families of transcription factors.

**Figure S2.** Mutant phenotypes.

**Figure S3.** Expression of *TCP14* and *TCP15* in stamens.

**Figure S4.** *TCP14* and *TCP15* expression levels in an array of tissues and organs.

**Figure S5.** Combined changes to leaf shape along PC2 and PC4.

Please note: As a service to our authors and readers, this journal provides supporting information supplied by the authors. Such materials are peer-reviewed and may be re-organized for online delivery, but are not copy-edited or typeset. Technical support issues arising from supporting information (other than missing files) should be addressed to the authors.

## References

[b1] Aguilar-Martínez JA, Poza-Carrión C, Cubas P (2007). Arabidopsis *BRANCHED1* acts as an integrator of branching signals within axillary buds. Plant Cell.

[b2] Alonso JM, Stepanova AN (2003). T-DNA mutagenesis in Arabidopsis. Methods Mol. Biol..

[b3] Bemis SM, Torii KU (2007). Autonomy of cell proliferation and developmental programs during Arabidopsis aboveground organ morphogenesis. Dev. Biol..

[b4] Bouché N, Bouchez D (2001). Arabidopsis gene knockout: phenotypes wanted. Curr. Opin. Plant Biol..

[b5] Boudolf V, Vlieghe K, Beemster GT, Magyar Z, Torres Acosta JA, Maes S, Van Der Schueren E, Inzé D, De Veylder L (2004). The plant-specific cyclin-dependent kinase CDKB1;1 and transcription factor E2Fa-DPa control the balance of mitotically dividing and endoreduplicating cells in Arabidopsis. Plant Cell.

[b6] Breuil-Broyer S, Morel P, de Almeida-Engler J, Coustham V, Negrutiu I, Trehin C (2004). High-resolution boundary analysis during *Arabidopsis thaliana* flower development. Plant J..

[b7] Clough SJ, Bent AF (1998). Floral dip: a simplified method for *Agrobacterium*-mediated transformation of *Arabidopsis thaliana*. Plant J..

[b8] Cubas P, Lauter N, Doebley J, Coen E (1999). The TCP domain: a motif found in proteins regulating plant growth and development. Plant J..

[b9] De Veylder L, Beeckman T, Beemster GT (2002). Control of proliferation, endoreduplication and differentiation by the Arabidopsis E2Fa-DPa transcription factor. EMBO J..

[b10] Dinneny JR, Yadegari R, Fischer RL, Yanofsky MF, Weigel D (2004). The role of JAGGED in shaping lateral organs. Development.

[b11] Doebley J, Stec A, Hubbard L (1997). The evolution of apical dominance in maize. Nature.

[b12] Donnelly PM, Bonetta D, Tsukaya H, Dengler RE, Dengler NG (1999). Cell cycling and cell enlargement in developing leaves of Arabidopsis. Dev. Biol..

[b13] Efroni I, Blum E, Goldschmidt A, Eshed Y (2008). A protracted and dynamic maturation schedule underlies Arabidopsis leaf development. Plant Cell.

[b14] Finlayson SA (2007). Arabidopsis TEOSINTE BRANCHED1-LIKE 1 regulates axillary bud outgrowth and is homologous to monocot TEOSINTE BRANCHED1. Plant Cell Physiol..

[b15] Gaudin V, Lunness PA, Fobert PR, Towers M, Riou-Khamlichi C, Murray JA, Coen E, Doonan JH (2000). The expression of D-cyclin genes defines distinct developmental zones in snapdragon apical meristems and is locally regulated by the *Cycloidea* gene. Plant Physiol..

[b16] Giraud E, Ng S, Carrie C, Duncan O, Low J, Lee CP, Van Aken O, Millar A, Murcha M, Whelan J (2010). TCP transcription factors link the regulation of genes encoding mitochondrial proteins with the circadian clock in *Arabidopsis thaliana*. Plant Cell.

[b17] Granier C, Tardieu F (2009). Multi-scale phenotyping of leaf expansion in response to environmental changes: the whole is more than the sum of parts. Plant Cell Environ..

[b18] Guo Z, Fujioka S, Blancaflor EB, Miao S, Gou X, Li J (2010). *TCP1* modulates brassinosteroid biosynthesis by regulating the expression of the key biosynthetic gene *DWARF4* in *Arabidopsis thaliana*. Plant Cell.

[b19] Hellens RP, Edwards A, Leyland NR, Bean S, Mullineaux PM (2000). pGreen: a versatile and flexible binary Ti vector for *Agrobacterium*-mediated plant transformation. Plant Mol. Biol..

[b20] Hervé C, Dabos P, Bardet C, Jauneau A, Auriac MC, Ramboer A, Lacout F, Tremousaygue D (2009). *In vivo* interference with *AtTCP20* function induces severe plant growth alterations and deregulates the expression of many genes important for development. Plant Physiol..

[b21] Hiratsu K, Matsui K, Koyama T, Ohme-Takagi M (2003). Dominant repression of target genes by chimeric repressors that include the EAR motif, a repression domain, in Arabidopsis. Plant J..

[b22] Ingram GC, Waites R (2006). Keeping it together: co-ordinating plant growth. Curr. Opin. Plant Biol..

[b23] Jefferson RA, Kavanagh TA, Bevan MW (1987). GUS fusions: β-glucuronidase as a sensitive and versatile gene fusion marker in higher plants. EMBO J..

[b24] Kieffer M, Stern Y, Cook H, Clerici E, Maulbetsch C, Laux T, Davies B (2006). Analysis of the transcription factor WUSCHEL and its functional homologue in *Antirrhinum* reveals a potential mechanism for their roles in meristem maintenance. Plant Cell.

[b25] Kim GT, Tsukaya H, Uchimiya H (1998). The *CURLY LEAF* gene controls both division and elongation of cells during the expansion of the leaf blade in *Arabidopsis thaliana*. Planta.

[b26] Kosugi S, Ohashi Y (1997). PCF1 and PCF2 specifically bind to *cis* elements in the rice proliferating cell nuclear antigen gene. Plant Cell.

[b27] Koyama T, Furutani M, Tasaka M, Ohme-Takagi M (2007). TCP transcription factors control the morphology of shoot lateral organs via negative regulation of the expression of boundary-specific genes in Arabidopsis. Plant Cell.

[b28] Koyama T, Sato F, Ohme-Takagi M (2010a). A role of *TCP1* in the longitudinal elongation of leaves in Arabidopsis. Biosci. Biotechnol. Biochem..

[b29] Koyama T, Mitsuda N, Seki M, Shinozaki K, Ohme-Takagi M (2010b). TCP transcription factors regulate the activities of *ASYMMETRIC LEAVES1* and miR164, as well as the auxin response, during differentiation of leaves in Arabidopsis. Plant Cell.

[b30] Krysan PJ, Young JC, Sussman MR (1999). T-DNA as an insertional mutagen in Arabidopsis. Plant Cell.

[b31] Laufs P, Peaucelle A, Morin H, Traas J (2004). MicroRNA regulation of the *CUC* genes is required for boundary size control in Arabidopsis meristems. Development.

[b32] Li C, Potuschak T, Colón-Carmona A, Gutiérrez RA, Doerner P (2005). Arabidopsis *TCP20* links regulation of growth and cell division control pathways. Proc. Natl Acad. Sci. USA.

[b33] Luo D, Carpenter R, Vincent C, Copsey L, Coen E (1996). Origin of floral asymmetry in *Antirrhinum*. Nature.

[b34] Luo D, Carpenter R, Copsey L, Vincent C, Clark J, Coen E (1999). Control of organ asymmetry in flowers of *Antirrhinum*. Cell.

[b35] Martín-Trillo M, Cubas P (2010). TCP genes: a family snapshot ten years later. Trends Plant Sci..

[b36] Menges M, de Jager SM, Gruissem W, Murray JA (2005). Global analysis of the core cell cycle regulators of Arabidopsis identifies novel genes, reveals multiple and highly specific profiles of expression and provides a coherent model for plant cell cycle control. Plant J..

[b37] Nath U, Crawford BC, Carpenter R, Coen E (2003). Genetic control of surface curvature. Science.

[b38] Navaud O, Dabos P, Carnus E, Tremousaygue D, Hervé C (2007). TCP transcription factors predate the emergence of land plants. J. Mol. Evol..

[b39] Pagnussat GC, Yu HJ, Ngo QA, Rajani S, Mayalagu S, Johnson CS, Capron A, Xie LF, Ye D, Sundaresan V (2005). Genetic and molecular identification of genes required for female gametophyte development and function in Arabidopsis. Development.

[b40] Palatnik JF, Allen E, Wu X, Schommer C, Schwab R, Carrington JC, Weigel D (2003). Control of leaf morphogenesis by microRNAs. Nature.

[b41] Pruneda-Paz JL, Breton G, Para A, Kay SA (2009). A functional genomics approach reveals *CHE* as a component of the Arabidopsis circadian clock. Science.

[b42] Ramirez-Parra E, Desvoyes B, Gutierrez C (2005). Balance between cell division and differentiation during plant development. Int. J. Dev. Biol..

[b43] Schellmann S, Hülskamp M (2005). Epidermal differentiation: trichomes in Arabidopsis as a model system. Int. J. Dev. Biol..

[b44] Schommer C, Palatnik JF, Aggarwal P, Chételat A, Cubas P, Farmer EE, Nath U, Weigel D (2008). Control of jasmonate biosynthesis and senescence by miR319 targets. PLoS Biol..

[b45] Serrano-Cartagena J, Candela H, Robles P, Ponce MR, Pérez-Pérez JM, Piqueras P, Micol JL (2000). Genetic analysis of *incurvata* mutants reveals three independent genetic operations at work in Arabidopsis leaf morphogenesis. Genetics.

[b46] Sessions A, Burke E, Presting G (2002). A high-throughput Arabidopsis reverse genetics system. Plant Cell.

[b47] Swofford D (2002). Paup*: Phylogenetic Analysis Using Parsimony.

[b48] Takeda T, Amano K, Ohto MA, Nakamura K, Sato S, Kato T, Tabata S, Ueguchi C (2006). RNA interference of the Arabidopsis putative transcription factor *TCP16* gene results in abortion of early pollen development. Plant Mol. Biol..

[b49] Tatematsu K, Ward S, Leyser O, Kamiya Y, Nambara E (2005). Identification of *cis*-elements that regulate gene expression during initiation of axillary bud outgrowth in Arabidopsis. Plant Physiol..

[b50] Tatematsu K, Nakabayashi K, Kamiya Y, Nambara E (2008a). Transcription factor *AtTCP14* regulates embryonic growth potential during seed germination in *Arabidopsis thaliana*. Plant J..

[b51] Tatematsu K, Kamiya Y, Nambara E (2008b). Co-regulation of ribosomal protein genes as an indicator of growth status: comparative transcriptome analysis on axillary shoots and seeds in Arabidopsis. Plant Signal. Behav..

[b52] Trémousaygue D, Garnier L, Bardet C, Dabos P, Hervé C, Lescure B (2003). Internal telomeric repeats and ‘TCP domain’ protein-binding sites co-operate to regulate gene expression in *Arabidopsis thaliana* cycling cells. Plant J..

[b53] Weight C, Parnham D, Waites R (2008). LeafAnalyser: a computational method for rapid and large-scale analyses of leaf shape variation. Plant J..

[b54] Weir I, Lu J, Cook H, Causier B, Schwarz-Sommer Z, Davies B (2004). *CUPULIFORMIS* establishes lateral organ boundaries in *Antirrhinum*. Development.

[b55] Welchen E, Gonzalez DH (2006). Overrepresentation of elements recognized by TCP-domain transcription factors in the upstream regions of nuclear genes encoding components of the mitochondrial oxidative phosphorylation machinery. Plant Physiol..

[b56] Yao X, Ma H, Jian Wang J, Zhang D (2007). Genome-wide comparative analysis and expression pattern of *TCP* gene families in *Arabidopsis thaliana* and *Oryza sativa*. J. Integr. Plant Biol..

[b57] Zimmermann P, Hirsch-Hoffmann M, Hennig L, Gruissem W (2004). GENEVESTIGATOR. Arabidopsis microarray database and analysis toolbox. Plant Physiol..

