# Supplementary figures and images for: TCP14 and TCP15 affect internode length and leaf shape in Arabidopsis
Source: Plant J. 2011 Jul 21;68(1):147–58. doi: 10.1111/j.1365-313X.2011.04674.x (PMC3229714; doi:10.1111/j.1365-313X.2011.04674.x)

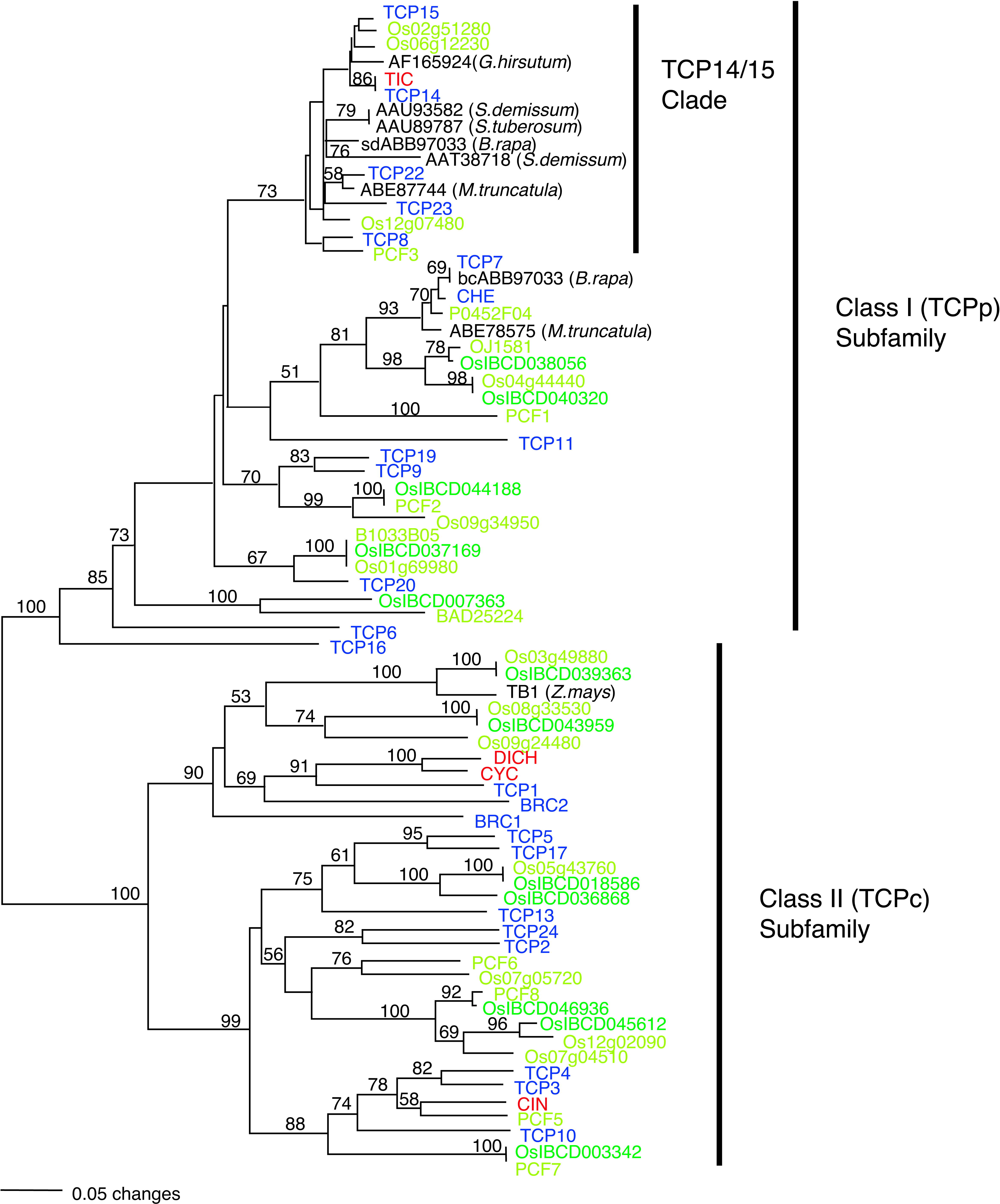

Supplement: Supplementary file 1 [file tpj0068-0147-SD1.tif]

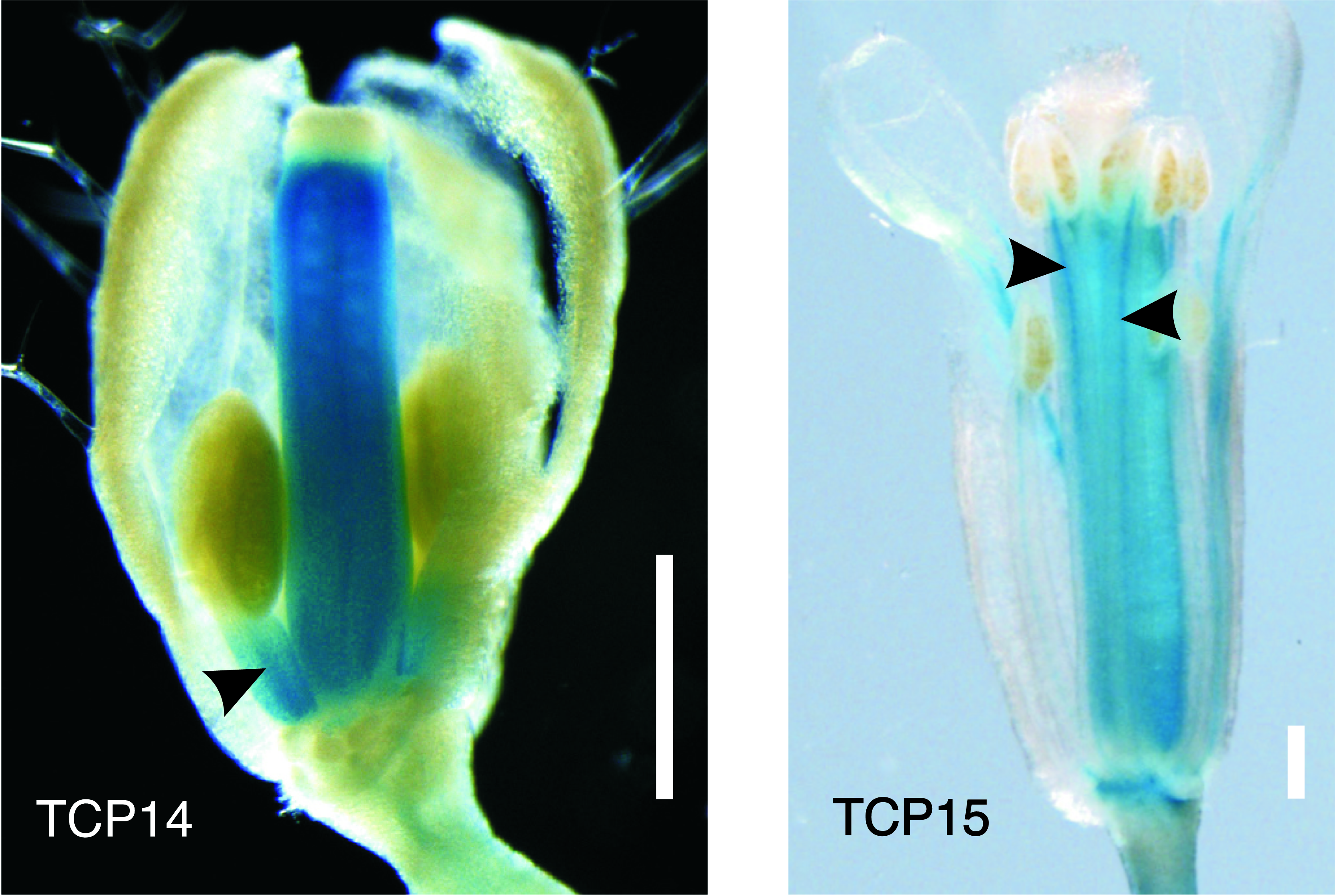

Supplement: Supplementary file 3 [file tpj0068-0147-SD3.tif]

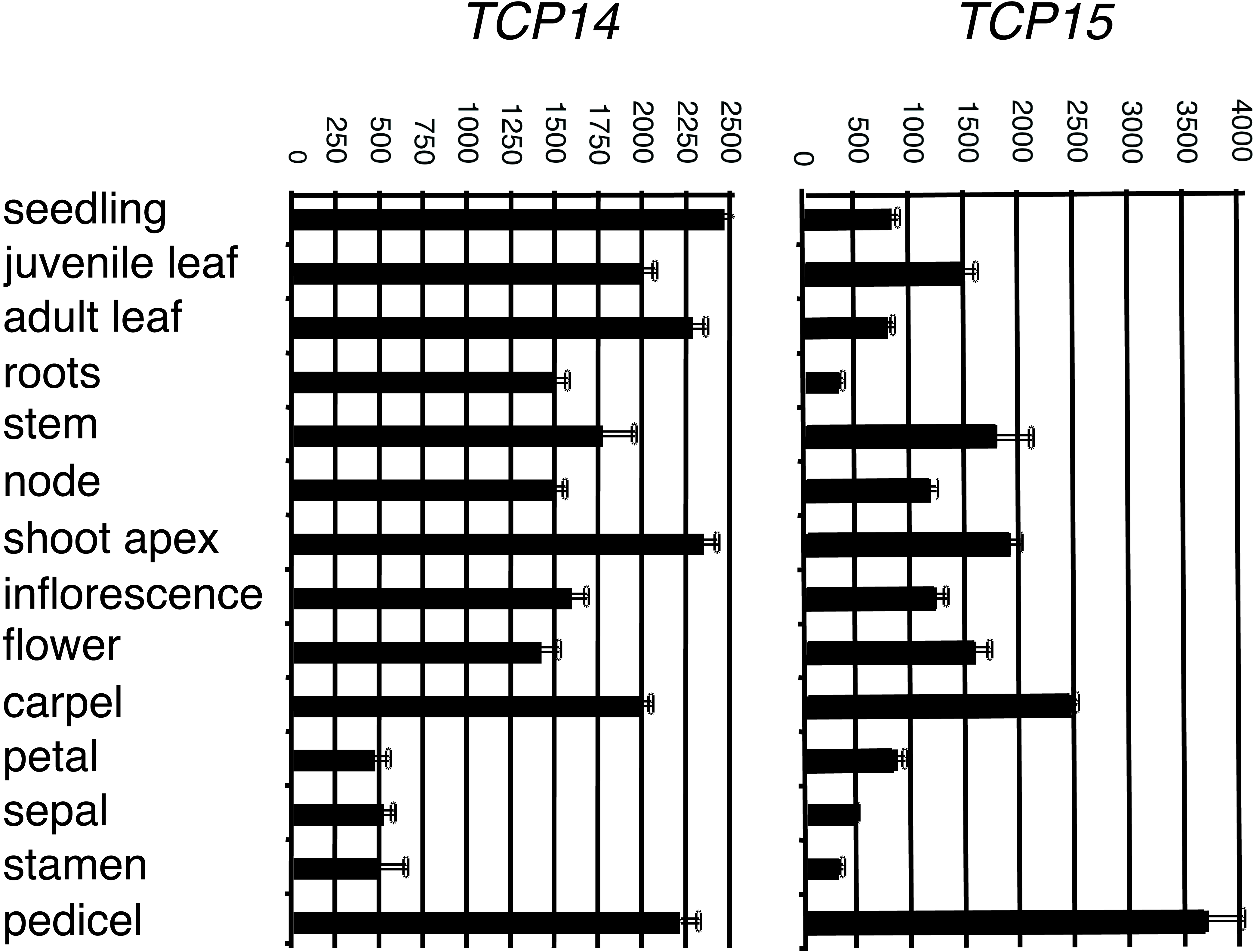

Supplement: Supplementary file 4 [file tpj0068-0147-SD4.tif]

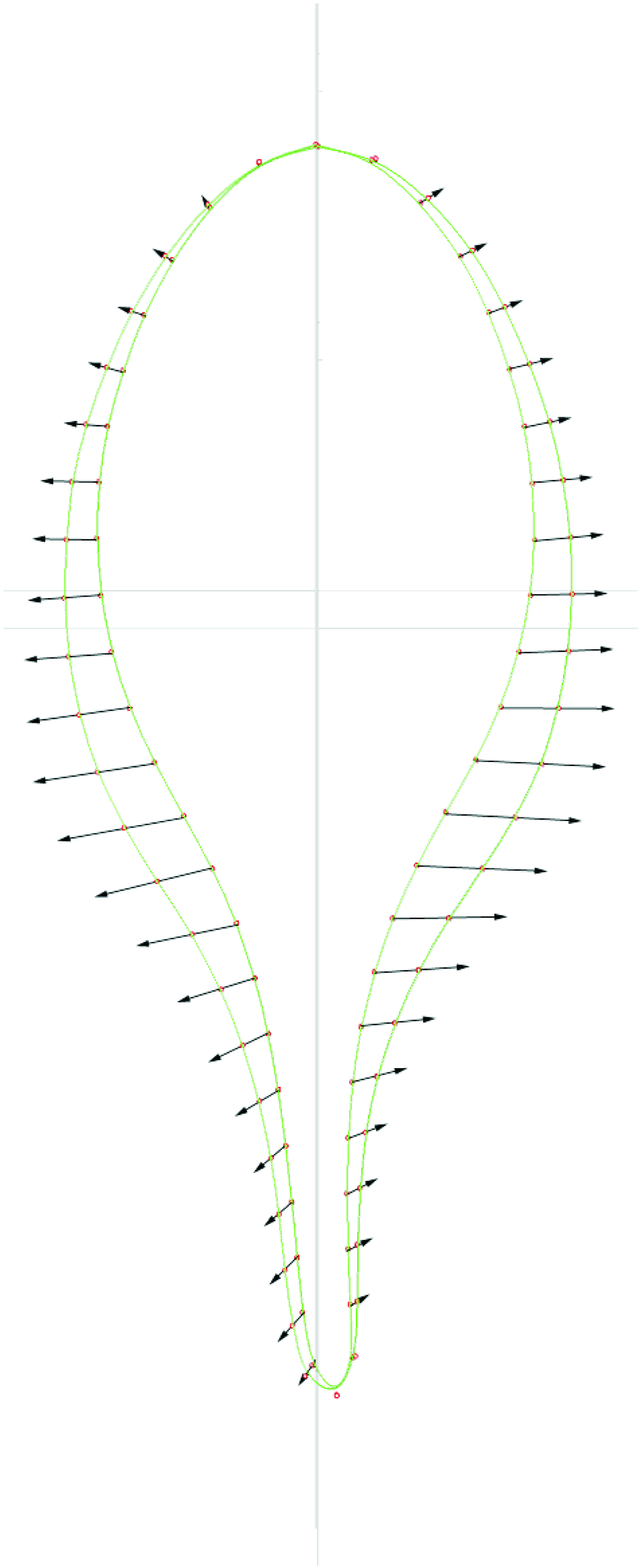

Supplement: Supplementary file 5 [file tpj0068-0147-SD5.tif]
