# Supplementary material for: TCP14 and TCP15 affect internode length and leaf shape in Arabidopsis
Source: Plant J. 2011 Jul 21;68(1):147–58. doi: 10.1111/j.1365-313X.2011.04674.x (PMC3229714; doi:10.1111/j.1365-313X.2011.04674.x)
Supplement: Supplementary file 6 [file tpj0068-0147-SD6.doc]

**Fig. S1.** Phylogenetic analysis of the *Arabidopsis* and rice TCP family of transcription factors. NJ analysis of the TCP DNA binding domain together with the adjacent CC domain using PAUP4.0b (Swofford, 2002). Branches with bootstrap (1000 replicates) support > 50% are indicated. *Arabidopsis* TCP proteins (blue) as Cubas (2000); rice (*O.sativa ssp. Japonica* proteins (light green), *O.sativa ssp. indica* proteins (dark green));  *Antirrhinum* proteins (red) TIC (AJ580844), CIN (AA043102), CYC (AAP84126), DICH (ABI26243). Selected genes form other species are shown in black.

**Fig. S2**. Mutant phenotype. (A) Mean inflorescence height of four different *tcp14tcp15* double mutant allelic combinations (white bars) and WT inflorescence in respective backgrounds (black bars) (30<n<170). (B-D) Complementation of *tcp14-4 tcp15-3* in lines transformed with the *pTCP14:TCP14* construct: (B) Control double mutant (left) and complemented line (right) (36 days old), (C) Mature silique pedicel length (n=100) and (D) inflorescence height (n=20) in double mutant (white bar ), complemented lines (grey bar), and WT control (black bar). (mean, bar = SD). Letters above bars represent statistically significant different means (Student’s *t*-test, p<0.001).

**Fig. S3.** Expression of *TCP14* and *TCP15* in stamens. Left: *TCP14* (*pTCP14:TCP14:GUS*) in stage 8 flower. Right: TCP15 (*pTCP15:TCP15:GUS*) in stage 15 flower. Arrowheads show staining in filaments of the vascular system. (Bars: 500 μm).

**Fig. S4**. Compilation of *TCP14* and *TCP15* expression levels in an array of tissues and organs obtained using the GENEVESTIGATOR (Zimmermann et al., 2004) microarray database.

**Fig. S5**. Combined changes to leaf shape along PC2 and PC4. Leaf point models for *Arabidopsis* leaves. The centre most leaf model is the mean leaf from 1500 leaves used in this analysis. Underlying the mean leaf is a second leaf that represents a leaf +1 SD along PC2 and -1 SD along PC4 from the mean. This shift to a higher PC2 value and a lower PC4 value also occurs in *tcp14-4*, *tcp15-3* and *tcp14-4 tcp15-3*. The change in shape generated by altering PC2 and PC4 values represent the modifications to leaf that occur in the absence of *TCP14* and *TCP15*. The black arrows indicate the direction in which each landmark moves with the changes to PC values. The arrows tips finish at a position that corresponds to a +2 SD shift along PC2 and -2SD shift along PC4.
